# BaCo_0.06_Bi_0.94_O_3_-Doped NiZn Ferrites for High Frequency Low Loss Current Sensors: LTCC Sintering and Magnetic Properties

**DOI:** 10.3390/s25092731

**Published:** 2025-04-25

**Authors:** Shao-Pu Jiang, Chang-Lai Yuan, Wei Liu, Lin Li, Huan Li, Jing-Tai Zhao

**Affiliations:** Guangxi Key Laboratory of Information Material, School of Materials Science and Engineering, Guilin University of Electronic Technology, Guilin 541004, Chinalihuan@guet.edu.cn (H.L.)

**Keywords:** NiZn ferrites, current sensor, magnetic properties, LTCC technology

## Abstract

In order to meet the demand for high-frequency current sensors in 5G communication and new energy fields, there is an urgent need to develop high-performance nickel-zinc ferrite-based co-fired ceramic magnetic cores. In this study, a nickel-zinc ferrite core based on low temperature co-fired ceramic (LTCC) technology was developed. The regulation mechanism of BaCo_0.06_Bi_0.94_O_3_ doping on the low-temperature sintering characteristics of NiZn ferrites was systematically investigated. The results show that the introduction of BaCo_0.06_Bi_0.94_O_3_ reduces the sintering temperature to 900 °C and significantly improves the density and grain uniformity of ceramics. When the doping amount is 0.75 wt%, the sample exhibits the lowest coercivity of 35.61 Oe and the following optimal soft magnetic properties: initial permeability of 73.74 (at a frequency of 1 MHz) and quality factor of 19.64 (at a frequency of 1 MHz). The highest saturation magnetization reaches 66.07 emu/g at 1 wt% doping. The results show that BaCo_0.06_Bi_0.94_O_3_ doping can regulate the grain boundary liquid phase distribution and modulate the magnetocrystalline anisotropy, which provides an experimental basis and optimization strategy for the application of LTCC technology in high-frequency current sensors.

## 1. Introduction

The progress of modern electronic technology promotes the development of communication equipment in the direction of high frequency, miniaturization, and multi-function. As a key component in the communication system, the performance of the multi-layer co-fired device directly determines the overall efficiency of the system [1,2,3,4,5,6]. To meet these requirements, the selection of substrate materials is critical. Ferrite materials emerge as ideal candidates for high-frequency device substrates and sensor applications due to their moderate permeability, low loss, and high magnetization characteristics [7,8,9]. Among them, NiZn ferrites have shown significant advantages in high-frequency current sensors due to their high permeability, low magnetic loss, and excellent chemical stability.

However, the relatively high sintering temperature of NiZn ferrites imposes significant constraints on their application in low-temperature co-fired ceramic (LTCC) technology [10,11,12,13,14,15,16]. To achieve process compatibility with silver electrode co-firing, the sintering temperature must be strictly maintained below the melting point of silver (approximately 961 °C). Conventional NiZn ferrite systems typically exhibit microstructural defects such as inhomogeneous grain growth and insufficient densification under low-temperature sintering conditions, leading to substantial degradation of both magnetic and dielectric properties [17]. To address these technical challenges, researchers commonly employ the strategy of adding sintering aids (e.g., Bi_2_O_3_, B_2_O_3_) [18], aiming to achieve effective regulation of sintering temperature and optimization of material microstructure. Nevertheless, such additives may introduce non-magnetic ionic impurities, thereby adversely affecting the magnetic performance of the material.

In recent years, researchers have focused on enhancing the low-temperature sintering characteristics of NiZn ferrites through process optimization and the development of novel sintering aids. For instance, the preparation of ultrafine ferrite powder matrices through chemical synthesis techniques, including co-precipitation, hydrothermal processing, microwave heating, and sol-gel combustion, has proven effective in reducing sintering temperature. Furthermore, advanced sintering techniques such as spark plasma sintering, flash sintering, hot-press sintering, and microwave sintering have demonstrated significant potential in enhancing ferrite densification efficiency while reducing sintering temperature. Industrial production puts forward higher requirements for cost and efficiency. The research on oxide doping sintering technology is of great value. This technology can effectively reduce the sintering temperature of NiZn ferrites and significantly improve the material properties. Reducing the sintering temperature of NiZn ferrites is a key breakthrough for enabling its co-firing with silver electrodes. At present, many methods have been developed to reduce the sintering temperature of NiZn ferrites, such as adding low melting point oxide V_2_O_5_ [19]; at the same time, in order to improve the magnetic properties of NiCuZn ferrites, the methods of introducing additives such as MoO_3_-WO_3_ [20] and Bi_2_O_3_-Li_2_CO_3_ [21] have been explored.

BaBiO_3_ is a kind of ceramic material with special properties, which has a high dielectric constant, low loss factor, and good thermal stability, and is widely used in the field of electronics and optoelectronics. The excellent performance of BaBiO_3_ makes it an ideal choice to improve the performance of other materials, especially in magnetic materials, as an additive to enhance their performance. In NiZn ferrite materials, the addition of BaBiO_3_ as an additive can significantly improve the magnetic properties of the material, especially in the low-temperature sintering process. Because BaBiO_3_ has good dielectric and magnetic properties, it can improve the permeability, saturation magnetization, and temperature stability of NiZn ferrite by adjusting the lattice structure of NiZn ferrite and increasing the interaction between magnetic particles. In this way, BaBiO_3_ can help NiZn ferrites perform more stably in high-frequency applications, reduce electromagnetic losses, and improve overall performance. In addition, the addition of BaBiO_3_ can also promote the low-temperature sintering process of NiZn ferrite. BaBiO_3_ has high sintering activity, which can promote the sintering of NiZn ferrite at lower temperatures, thereby reducing energy consumption and increasing the density of the material. The incorporation of cobalt oxide mainly enhances the magnetism of the material by providing Co^2+^ ions. Co^2+^ ion has a high magnetic moment, so its addition can effectively improve the saturation magnetization and coercivity of NiZn ferrite. Co^2+^ ions can also promote the uniform growth of grains, thereby improving the magnetic domain structure of the material and improving its magnetic permeability and high-frequency performance. Therefore, we try to improve the properties of NiZn ferrite by adding the Co element into BaBiO_3_ as an additive.

The novel sintering aid BaCo_0.06_Bi_0.94_O_3_ demonstrates exceptional magnetic property regulation capabilities. The purpose of this paper is to investigate its effect as an additive on the low-temperature sintering properties of NiZn ferrites. By adjusting the doping ratio, the influence of BaCo_0.06_Bi_0.94_O_3_ on the microstructure and magnetic properties of the material was studied, and its application potential in LTCC technology and high-frequency laminated devices was explored. We expect to obtain NiZn ferrite materials with high permeability, low loss, and high magnetization by optimizing the additional amount of BaCo_0.06_Bi_0.94_O_3_, which provides a new solution for the miniaturization and high performance of electronic components. Through this study, we hope to provide a new idea for the low-temperature sintering technology of NiZn ferrite material and lay a theoretical foundation for its application in high-frequency current sensors.

## 2. Materials and Methods

### 2.1. Materials

NiZn ferrite ceramic samples were prepared using the oxide ceramic method. High-purity BaCo_0.06_Bi_0.94_O_3_ precursors (BaCO_3_, Co_2_O_3_, and Bi_2_O_3_, purity ≥99.99%, purchased from Aladdin, Shanghai, China) were precisely weighed and mixed with deionized water according to stoichiometric ratios, followed by 6-h planetary ball milling. The mixed powder was then pre-sintered in a muffle furnace at 725 °C for 2 h to complete solid-state reactions.

### 2.2. I Preparation of Composite Ceramic Samples

Commercial NiZn ferrite powder was blended with sintering aids ((1 − *x*)Ni_0.4_Zn_0.6_Fe_2_O_4_ + *x*BaCo_0.06_Bi_0.94_O_3_, 0.0 ≤ *x* ≤ 0.01). The mixed powder underwent 6-h wet ball milling with deionized water, followed by vacuum drying. Then, 8% weight polyvinyl alcohol (PVA) binder was added for spray granulation. The dried powder was uniaxially pressed at 20 MPa into standard toroidal green compacts (outer diameter 20 mm, inner diameter 10 mm, thickness 5 mm), followed by sintering at 900 °C for 1 h in an air atmosphere.

### 2.3. Characterization

Bulk density was determined by the Archimedes drainage method using a precision densitometer. Crystal structure analysis was performed with an Empyrean PIXcel3D X-ray (PANalytical, Almelo, The Netherlands) diffractometer, with diffraction data refined by Jade 6.5 software using the Rietveld method. Microstructural characterization and elemental distribution analysis were conducted using a QUANTA 450-FEG (FEI Company, Hillsboro, OR, USA) field-emission scanning electron microscope equipped with an energy-dispersive spectrometer (EDS, Oxford X-Max20, Oxford Instruments plc, Oxford, UK). Complex permeability spectra were measured by an Agilent E4991B (Agilent Technologies Inc., Santa Clara, CA, USA) impedance analyzer. The hysteresis loop is obtained by multiple measurements using the Lake Shore 7404-S (Lake Shore Cryotronics Inc., Westerville, OH, USA) vibrating sample magnetometer. X-ray photoelectron spectroscopy (XPS) was obtained by the ESCALAB 250 XI (Thermo Fisher Scientific Inc., Waltham, MA, USA) spectrometer.

## 3. Results and Discussion

### 3.1. Phase Analysis

**Figure 1:**
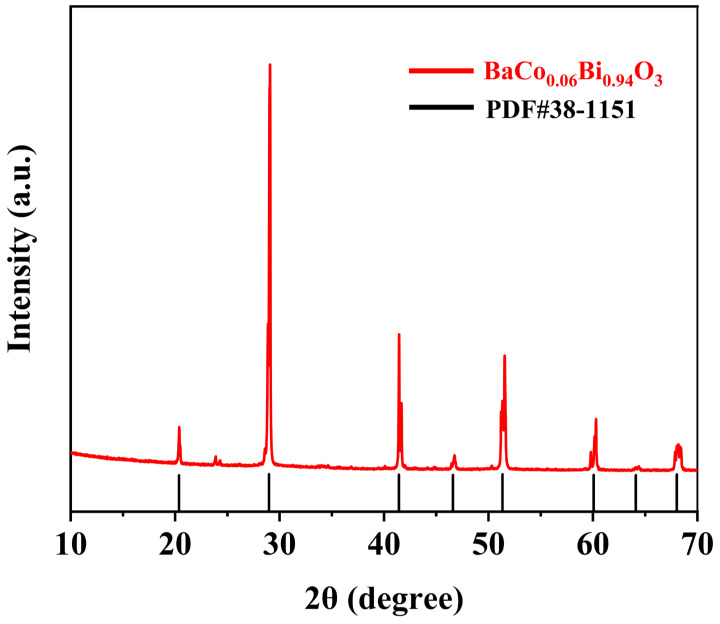
The XRD patterns of BaCo_0.06_Bi_0.94_O_3_.

**Figure 2:**
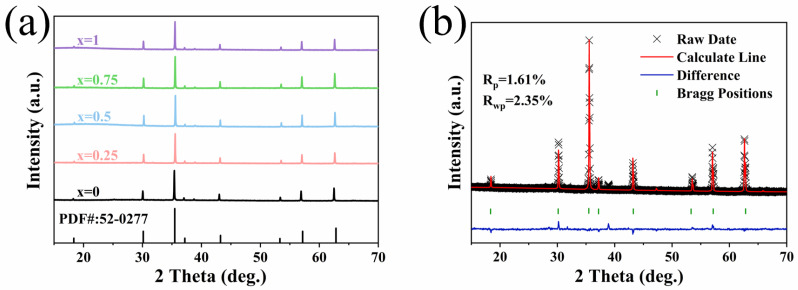
(**a**) The XRD patterns of ceramic samples doped with *x* wt% BaCo_0.06_Bi_0.94_O_3_ and (**b**) Rietveld refinement XRD results of ceramic samples doped with 0.75 wt% BaCo_0.06_Bi_0.94_O_3_.

Figure 1 shows the XRD pattern of BaCo_0.06_Bi_0.94_O_3_, and the diffraction peak of the sample corresponds well to JCPDS PDF#38-1151, which proves the successful synthesis of BaCo_0.06_Bi_0.94_O_3_. Figure 2a shows the XRD pattern of Ni_0.4_Zn_0.6_Fe_2_O_4_/BaCo_0.06_Bi_0.94_O_3_ composite ceramic samples. All samples exhibit a single spinel phase structure, where the primary diffraction peaks correspond to the (111), (022), (113), (222), (004), (224), (333), and (044) crystal planes, showing excellent agreement with the NiZn ferrites with reference JCPDS PDF#52-0277. Figure 2b shows the Rietveld refinement results of ceramic samples with a doping amount of 0.75 wt%. Through the refinement of XRD diffraction data, the Rietveld method was used to accurately fit the diffraction patterns of the samples. During the refinement process, the position, strength, and lattice parameters of each crystal phase in the sample were determined by analyzing the shape and strength of the diffraction peaks. The refinement results show that the crystal structure of the ceramic sample does not change significantly after adding 0.75 wt% BaCo_0.06_Bi_0.94_O_3_, and all diffraction peaks are still consistent with the expected NiZn ferrite and BaCo_0.06_Bi_0.94_O_3_, which proves the composite stability of the two materials. The refined calculation results further obtained the lattice constants of each phase and confirmed that the formation of NiZn ferrite phase was not affected by the addition of BaCo_0.06_Bi_0.94_O_3_, thus proving the successful synthesis of the composite material. The diffraction peak position did not change with the addition of BaCo_0.06_Bi_0.94_O_3_, indicating that the formation of the NiZn ferrites phase was not affected. XRD analysis confirms that the crystal structure of NiZn ferrites remains intact without phase transformation during sintering with this sintering aid.

**Figure 3:**
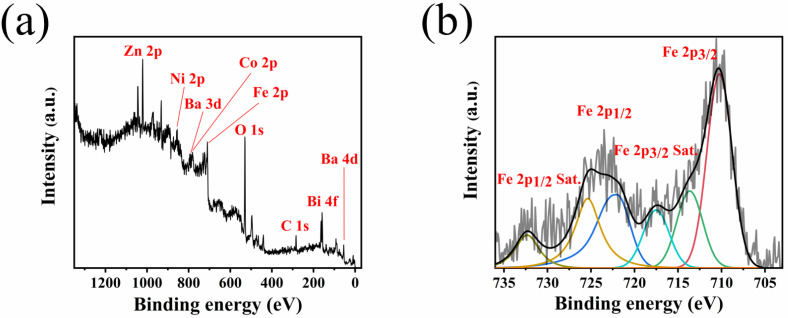
XPS spectra (**a**) and Fe high-resolution spectra (**b**) of NiZn ferrite ceramics with a BaCo_0.06_Bi_0.94_O_3_ doping ratio of 0.75 wt%.

The surface structure of the composite NiZn ferrite ceramics was characterized by XPS. As shown in Figure 3a, the characteristic peaks of Ba 3d, Co 2p, and Bi 4f can be clearly identified in the full spectrum of BaCo_0.06_Bi_0.94_O_3_ doped samples. XPS spectra combined with XRD patterns can further illustrate that the formation of the NiZn ferrite phase structure is not affected by additives or the successful introduction of additives. Figure 3b further demonstrates the high-resolution XPS spectra of 0.75 wt% BaCo_0.06_Bi_0.94_O_3_-doped NiZn ferrites sintered at 900 °C. The peak positions of Fe 2p and Fe 2p can reflect the valence state characteristics of iron ions: the sample shows a significant main peak of Fe 2p_3/2_ at 710.8 eV and 713.7 eV, Fe 2p_1/2_ at 725.2 eV and 722.2 eV, and a typical satellite peak appears near 719 eV, 732.5 eV, indicating that the iron element in the system exists in the form of Fe^3+^, and the characteristic signal of Fe^2+^ is not detected [22]. This result indicates that the electron transfer between Fe^3+^ and Fe^2+^+ is weak, which may be one of the important mechanisms for the low magnetic loss characteristics of the doped NiZn ferrite [23,24].

### 3.2. Microstructure Analysis

**Figure 4:**
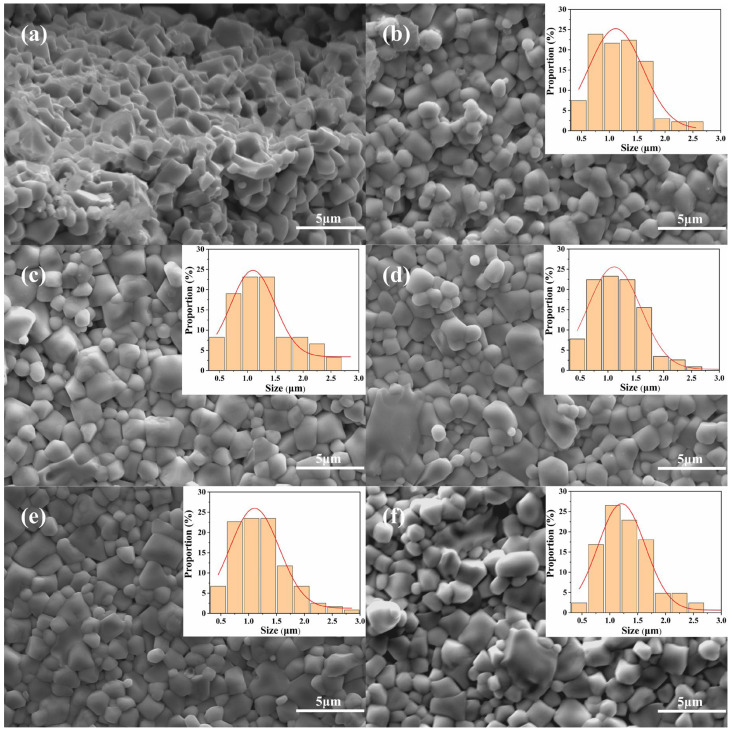
SEM images of (1 − *x*) Ni_0.4_Zn_0.6_Fe_2_O_4_ + *x* BaCo_0.06_Bi_0.94_O_3_ (0.0 ≤ *x* ≤ 0.01) composite ceramic samples: (**a**) *x* = 0, (**b**) *x* = 0, (**c**) *x* = 0.0025, (**d**) *x* = 0.005, (**e**) *x* = 0.0075, (**f**) *x* = 0.01.

Figure 4 shows the scanning electron microscopy (SEM) images of Ni_0.4_Zn_0.6_Fe_2_O_4_/BaCo_0.06_Bi_0.94_O_3_ composite ceramics sintered at 900 °C for 1 h. When the doping amount of BaCo_0.06_Bi_0.94_O_3_ is 0 wt%, the sintered body exhibits small grain sizes with larger size differences. The sample is composed of grain clusters showing a loose structure. The grains inside the clusters are in close contact to form grain boundaries, while there is less contact between the clusters, and there are a large number of interconnected pores (Figure 4a,b). This microstructure characteristic leads to low sample density. The main reason for its formation is the volume shrinkage caused by the density difference between the amorphous phase and the crystalline phase [25].

When the doping ratio of BaCo_0.06_Bi_0.94_O_3_ increases to 0.25 wt% and 0.75 wt%, the contact tightness between grains is significantly enhanced, the grain boundaries become clearer, and the number of pores is significantly reduced. At the same time, the morphology of the grains gradually tends to be regularized (Figure 4c,e) and the uniformity of the microstructure is improved, accompanied by an increase in the density of the sample. However, when the doping amount increases to 0.5 wt% and 1 wt%, abnormal grain growth and uneven pore distribution appear along the grain boundary region (Figure 4d,f), which may be the main reason for the sample density fluctuation. The increase of density may have an important influence on the improvement of magnetic properties.

When the doping ratio of BaCo_0.06_Bi_0.94_O_3_ increases to 1 wt%, dense small-sized grains and abnormally grown super-large grains appear in the grain boundary region (Figure 4f). Excessive doping-induced abnormal grain growth led to the formation of a supersaturated liquid phase at grain boundaries or within grains [26]. This liquid phase not only inhibited the normal growth of other grains but also caused an anomalous increase in average grain size while promoting intergranular pore formation and reducing material density. This phenomenon is the main factor that causes the magnetic properties to show the first enhancement and then deterioration, as shown in Table 1 ($M_{S}$). SEM analysis shows that the grain growth of Ni_0.4_Zn_0.6_Fe_2_O_4_ ferrite during sintering is significantly affected by BaCo_0.06_Bi_0.94_O_3_ doping. Combined with the experimental results, the appropriate amount of BaCo_0.06_Bi_0.94_O_3_ doping can reduce the sintering temperature of Ni_0.4_Zn_0.6_Fe_2_O_4_ ferrite to 900 °C.

### 3.3. Magnetic Properties

**Figure 5:**
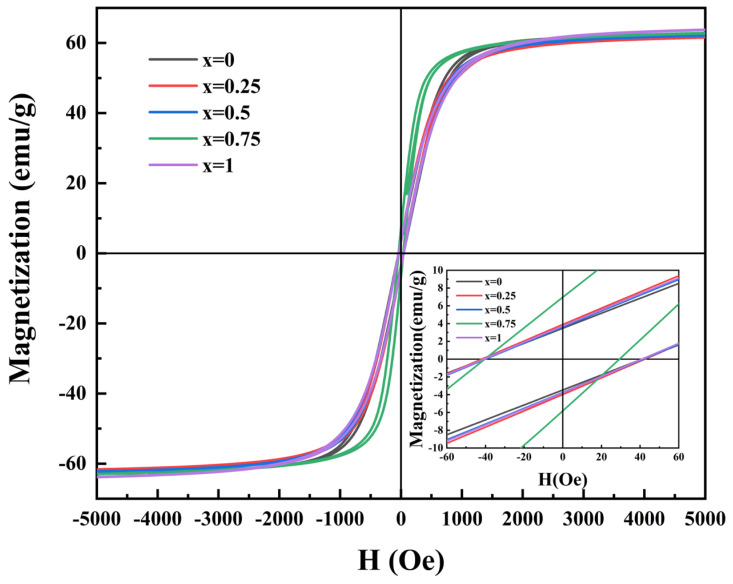
The magnetic hysteresis loops of ceramic samples doped with *x* wt% BaCo_0.06_Bi_0.94_O_3_.

Figure 5 displays the magnetic hysteresis loops of Ni_0.4_Zn_0.6_Fe_2_O_4_/BaCo_0.06_Bi_0.94_O_3_ composite ceramics at room temperature. The data in Table 1 show that the saturation magnetization of the Ni_0.4_Zn_0.6_Fe_2_O_4_/BaCo_0.06_Bi_0.94_O_3_ composite ceramic sample decreases slightly and then continues to rise when the doping ratio is 0.25 wt% compared with the undoped sample. All the samples show a typical hysteresis under the applied magnetic field and have low coercivity ($H_{C}$). When the BaCo_0.06_Bi_0.94_O_3_ doping concentration reached 0.75 wt%, the composite ceramic achieved magnetization saturation at a 600 Oe magnetic field while obtaining the minimum coercivity of 35.61 Oe. With the doping ratio increased to 1 wt%, the sample demonstrated the maximum saturation magnetization of 66.07 emu/g (Table 1). This enhancement of magnetization is essential to improve the signal-to-noise ratio of high-precision magnetic sensors because it is directly related to the detection sensitivity to magnetic flux. This enhancement in magnetization originated from the optimal addition of BaCo_0.06_Bi_0.94_O_3_, which improved grain size uniformity and increased sample density through reduced sintering temperature, thereby facilitating magnetization improvement. The improvement in saturation magnetization is due to the addition of BaCo_0.06_Bi_0.94_O_3_, which not only reduces the sintering temperature of Ni_0.4_Zn_0.6_Fe_2_O_4_ ferrite but also improves the uniformity of grain size and increases the density of the sample, thus promoting the increase in saturation magnetization. The scanning electron microscopy (SEM) microstructural analysis presented in Figure 4 further corroborated this mechanism.

The magnetism of spinel ferrite is mainly due to the difference in magnetic moments between A ($M_{A}$) and B ($M_{B}$) sublattices. The net magnetic moment ($M_{m}$) of the crystal can be defined as follows:

(1) $$M_{m}=M_{B}-M_{A}$$

When the doping content reached 0.25 wt%, the saturation magnetization ($M_{S}$) decreased to 63.79 emu/g. This phenomenon can be attributed to the excess nonmagnetic BaCo_0.06_Bi_0.94_O_3_. These ions (mainly Ba^2+^ and Bi^3+^) lead to the redistribution of metal cations at the B site by replacing Fe^3+^ ions. Such redistribution reduces the magnetic moment ($M_{B}$) of B-site lattice ions, consequently leading to the observed decline in saturation magnetization [27]. At a doping ratio of 0.75 wt%, the coercivity ($H_{C}$) of sintered samples reached a minimum value of 35.61 Oe. As described by Brown’s relation [28], coercivity exhibits a functional relationship with saturation magnetization ($M_{S}$), the anisotropy constant ($K_{1}$), and the magnetostriction coefficient ($\lambda_{S}$):

(2) $$H_{C}\propto \frac{\left|\lambda_{S}\right|\cdot \left|K_{1}\right|}{\mu_{0}M_{S}}$$

This relationship indicates that coercivity ($H_{C}$) is inversely proportional to saturation magnetization ($M_{S}$) and directly proportional to the magnetocrystalline anisotropy constant ($K_{1}$). As the BaCo_0.06_Bi_0.94_O_3_ content increases from 0 wt% to 0.5 wt%, the coercivity of the samples exhibits an initial increase followed by a decrease, which can be explained by the dominant effect of significantly reduced grain size on magnetocrystalline anisotropy [29,30]. When the BaCo_0.06_Bi_0.94_O_3_ content exceeds 0.75 wt%, the variation in coercivity is predominantly governed by saturation magnetization. The study reveals that 1 wt% BaCo_0.06_Bi_0.94_O_3_ represents the optimal doping concentration for enhancing the saturation magnetization of Ni_0.4_Zn_0.6_Fe_2_O_4_ ferrite, while 0.75 wt% BaCo_0.06_Bi_0.94_O_3_ enables the Ni_0.4_Zn_0.6_Fe_2_O_4_ ferrite to achieve the lowest coercivity value.

**Figure 6:**
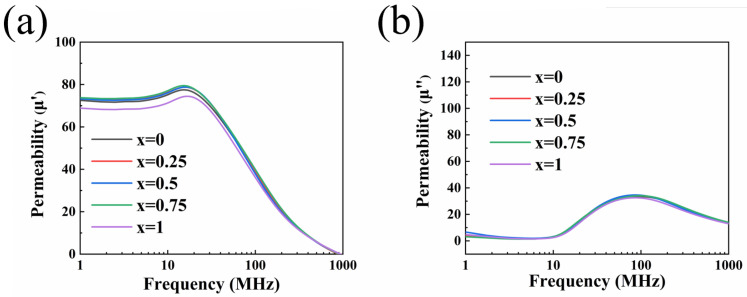
Complex permeability (**a**) real part and (**b**) imaginary part of ceramic samples doped with *x* wt% BaCo_0.06_Bi_0.94_O_3_.

Figure 6 demonstrates the frequency response characteristics of Ni_0.4_Zn_0.6_Fe_2_O_4_/BaCo_0.06_Bi_0.94_O_3_ composite ceramics after sintering at 900 °C for 1 h. Within the 1–1000 MHz frequency range, the real part of permeability (*μ*′) for all samples exhibited three-stage evolution characteristics: maintaining stable values in the low-frequency region (<10 MHz), showing a gradual increase in the mid-frequency region, and demonstrating a sharp decline in the high-frequency region. As seen in Figure 6a, a significant increase in the initial permeability and a decrease in the cut-off frequency are observed, which is consistent with Snoek’s law [31]. The results show that the appropriate amount of BaCo_0.06_Bi_0.94_O_3_ can significantly improve the permeability of the sample. When the doping amount is 0.75 wt%, the maximum initial permeability is about 73.74 (at a frequency of 1 MHz).

The experimental observations can be attributed to permeability evolution induced by distinct magnetization mechanisms. In the low-frequency range, the initial permeability can be expressed as complex permeability, which mainly comes from the two main magnetization mechanisms of spin rotation and magnetic domain wall motion [32,33]:

(3) $$\mu =1+\chi_{spin}+\chi_{dw}$$

where $\chi_{spin\;}$ and $\chi_{dw}$ represent the magnetic susceptibility corresponding to spin rotation and domain wall motion, respectively. For multi-domain samples, the magnetization mechanism is predominantly governed by domain wall motion [34]. Accordingly, Equation (3) can be rewritten as

(4) $$\mu \approx 1+\chi_{dw}=1+\frac{3}{16}\cdot M_{S}^{2}\cdot D/\gamma_{w}$$

In the formula, $M_{S}$ is the saturation magnetization, *D* is the average grain size, and $\gamma_{w}$ is the domain wall energy. Van der Zaag reported that when the size of NiZn ferrites is less than 2 µm, the grains reach a single domain state [35]. As shown in Table 1, the grains in the ferrites we prepared are determined to be single-domain states, and the main contribution mechanism of dynamic magnetization changes from domain wall motion to spin rotation magnetization mechanism. Based on this analysis, Equation (3) can be simplified to the following expression [36,37]:

(5) $$\mu \approx 1+\chi_{spin}=1+\frac{4\pi M_{S}}{H_{d}+H_{A}}$$

Here, $M_{S}$, $H_{d}$, and $H_{A}$ denote saturation magnetization, demagnetizing field, and magnetocrystalline anisotropy field, respectively. The increase in the permeability of the 0.75 wt% doped sample can be explained by the decrease in $H_{d}$ and the increase in $M_{S}$ caused by the increase in sample density. The results show that the Ni_0.4_Zn_0.6_Fe_2_O_4_ ferrite doped with 0.75 wt% BaCo_0.06_Bi_0.94_O_3_ has the highest permeability in the frequency range of 1~10 MHz.

Low magnetic loss is an important parameter for high-performance NiZn ferrites. BaCo_0.06_Bi_0.94_O_3_ can effectively reduce the magnetic loss of NiZn ferrites and achieve saturation magnetization characteristics at low applied magnetic field strength. The mechanism of action is derived from the optimal regulation of the microstructure of the material.

**Figure 7:**
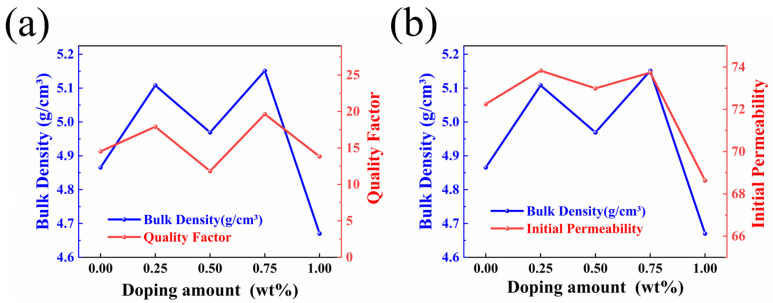
The (**a**) quality factor and initial (**b**) permeability of Ni_0.4_Zn_0.6_Fe_2_O_4_/BaCo_0.06_Bi_0.94_O_3_ composite ceramics change with density.

The quality factor is an important parameter to measure the loss characteristics of ferrite materials under an AC magnetic field, which is usually used to evaluate the energy loss and performance of materials. Figure 7a shows the fluctuation curves of the quality factor and density of the samples with the doping ratio of the additives. For ferrites, especially soft magnetic ferrites for high frequency applications, the quality factor is usually calculated by the following formula:

(6) $$Q=\frac{\mu^{\prime }}{\mu^{\prime \prime }}$$

**Figure 8:**
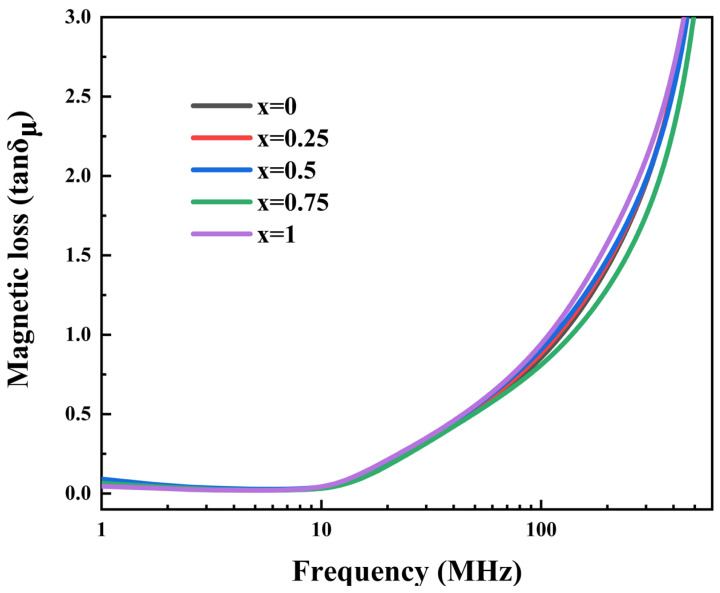
The magnetic loss of ceramic samples doped with *x* wt% BaCo_0.06_Bi_0.94_O_3_ in the frequency range of 1 MHz~1 GHz was measured.

Figure 8 shows the magnetic loss of Ni_0.4_Zn_0.6_Fe_2_O_4_/BaCo_0.06_Bi_0.94_O_3_ composite ceramics sintered at 900 °C in the frequency range of 1 MHz~1 GHz. The magnetic loss characteristics can be quantitatively described by the following mathematical expression:

(7) $$tan\delta_{\mu }=\frac{\mu^{\prime \prime }}{\mu^{\prime }}$$

Undoped samples exhibited a relatively low-quality factor at 1 MHz. The maximum Q value of 19.64 (at a frequency of 1 MHz) was achieved when the doping concentration of BaCo_0.06_Bi_0.94_O_3_ reached 0.75 wt%. As shown in Figure 7a, both sample density and quality factor followed consistent variation patterns with increasing BaCo_0.06_Bi_0.94_O_3_ content, demonstrating that densification plays a critical role in enhancing the quality factor. As shown in Figure 7a,b, with the change in BaCo_0.06_Bi_0.94_O_3_ doping concentration, the density, quality factor, and initial permeability of Ni_0.4_Zn_0.6_Fe_2_O_4_ ferrite show a synergistic change rule. The material density significantly affects the change trend of the initial permeability and quality factor. The experimental results show that BaCo_0.06_Bi_0.94_O_3_ doping can effectively regulate the material properties in the 1–10 MHz frequency band: the magnetic loss is significantly reduced, and the initial permeability and quality factor are improved.

**Figure 9:**
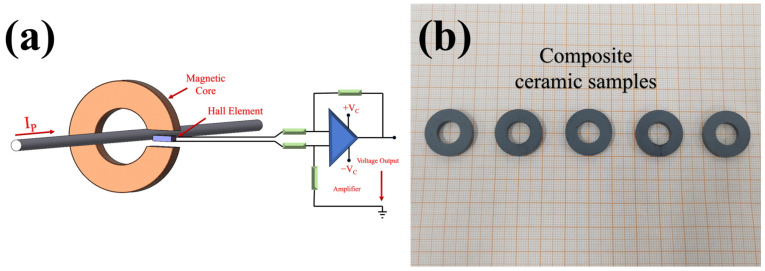
(**a**) Application of the magnetic core in the Hall sensor and (**b**) composite ceramic samples.

Figure 9a is the structure diagram of Hall current sensor. After integrating the optimized magnetic ring (Figure 9b) into the above current sensor, the high permeability, low coercivity, high frequency, low loss, high density, and other characteristics of the magnetic ring can provide sensitivity improvement, dynamic response acceleration, noise suppression, reliability enhancement, and other multiple advantages for the Hall current sensor [38]. According to the magnetic circuit model of the magnetic core in the Hall current sensor, the output magnetic induction intensity B is closely related to the initial permeability *μ* and the saturation magnetization M of the material. By introducing Ba impurity, the density of the NiZn ferrite core increases from 4.87 g/cm^3^ to 5.15 g/cm^3^, the microstructure is more compact, the porosity is reduced, and the magnetic flux conduction ability is effectively enhanced. The experimental results show that the initial permeability of the material increases from 72.25 to 73.83, an increase of about 2.2%. According to the formula

(8) $$V_{H}\propto \mu_{i}$$

It can be seen that the corresponding Hall output voltage will also be positively correlated, which will help to improve the sensitivity of the sensor. The formula shows that the material can still maintain a linear response under higher current conditions, which expands the linear working range of the sensor and improves its adaptability to high current signals. In summary, the density improvement and magnetic performance optimization achieved by doping control show consistency in both theoretical models and experimental data, which significantly enhances the comprehensive performance of the magnetic core in the application of Hall current sensors. In the future, multi-gap core design or gradient doping strategy can be further explored to meet the needs of 5G communication and miniaturized current sensors for high frequency and integration.

(9) $$B_{max}=\mu_{0}M_{s}$$

(10) $$I_{max}\propto M_{s}$$

## 4. Conclusions

In this paper, the effect of BaCo_0.06_Bi_0.94_O_3_ doping on the low-temperature sintering properties of Ni_0.4_Zn_0.6_Fe_2_O_4_ ferrite was systematically studied. The results show that proper doping can significantly reduce the sintering temperature of Ni_0.4_Zn_0.6_Fe_2_O_4_ ferrite to 900 °C and optimize its microstructure and magnetic properties. The sample with 0.75 wt% doping exhibited optimal comprehensive performance: uniform grain size (1.21 μm), minimum coercivity (35.61 Oe), highest quality factor (19.64 at a frequency of 1 MHz), and permeability (73.74 at a frequency of 1 MHz), along with significantly reduced magnetic losses. Excessive doping (≥0.75 wt%) induced abnormal grain growth and increased porosity, resulting in notable deterioration of magnetic properties. XRD and SEM analysis confirmed that the doping did not change the main phase structure of spinel. The magnetocrystalline anisotropy is affected by the liquid phase distribution at the grain boundary. The research results provide theoretical support for the development of high-frequency and low-loss LTCC components in current sensors, and 1 wt% and 0.75 wt% are established as the optimal doping thresholds for improving saturation magnetization (66.07 emu/g) and optimizing dynamic magnetic properties, respectively.

## Figures and Tables

**Table 1:** The performance parameters of Ni_0.4_Zn_0.6_Fe_2_O_4_ ferrite with BaCo_0.06_Bi_0.94_O_3_ addition sintered at 900 °C.

BaCo_0.06_Bi_0.94_O_3_ (wt%)	*μ_i_′* (1 MHz)	*D* (µm)	*M_S_* (emu/g)	*H_c_* (Oe)	*d* (g/cm^3^)	*Q* (1 MHz)
0	72.25	1.27	64.16	40.75	4.87	14.53
0.25	73.83	1.38	63.79	41.83	5.11	17.91
0.5	72.99	1.21	64.32	41.25	4.97	11.83
0.75	73.74	1.21	65.21	35.61	5.15	19.64
1	68.63	1.33	66.07	40.81	4.67	13.83

## Data Availability

The data that support the findings of this study are available from the corresponding author, upon reasonable request.
